# Identification of a novel locus *C*_*2*_ controlling canary yellow flesh color in watermelons

**DOI:** 10.3389/fgene.2023.1256627

**Published:** 2023-09-19

**Authors:** Girim Park, Durre Shahwar, Gaeun Jang, Jagyeong Shin, Gibeom Kwon, Younjae Kim, Chang Oh Hong, Bingkui Jin, Hoytaek Kim, Oakjin Lee, Younghoon Park

**Affiliations:** ^1^ Department of Horticultural Bioscience, Pusan National University, Miryang, Republic of Korea; ^2^ Partner Seeds Co., Ltd., Gimje, Republic of Korea; ^3^ Department of Life Science and Environmental Biochemistry, Pusan National University, Miryang, Republic of Korea; ^4^ Life and Industry Convergence Research Institute, Pusan National University, Miryang, Republic of Korea; ^5^ UNELL Biotechnology Co., Ltd., Weifang, China; ^6^ Department of Horticulture, Sunchon National University, Sunchon, Republic of Korea; ^7^ National Institute of Horticultural and Herbal Science, Rural Development Administration, Wanju, Republic of Korea

**Keywords:** *Citrullus lanatus*, flesh color, pentatricopeptide repeat, marker-assisted selection, watermelon

## Abstract

The flesh color of watermelon is an important trait that is determined by carotenoid composition and affects consumers’ fruit desirability. Although a complete dominant control by *C* locus (*Cllcyb*) for canary yellow flesh (CY) over red flesh has been reported, red and CY colors frequently appear as a mixed pattern in the same flesh (incomplete canary yellow, ICY) in F_1_ and inbred lines carrying dominant *C* alleles. Therefore, we examined the genetic control of the mixed color pattern in ICY using whole-genome resequencing of three ICY (ICY group) and three CY inbred lines (CY group), as well as genetic linkage mapping of an F_2_ population. The segregation pattern in 135 F_2_ plants indicated that CY is controlled by a single locus (named *C*_*2*_) dominant over ICY. The whole-genome resequencing of ICY and CY inbred lines revealed an ICY/CY-specific region of approximately 27.60–27.88 Mb on Chr. 2 that was polymorphic between the ICY and CY groups. Our genetic map, using nine cleaved amplified polymorphic sequence markers developed based on the single-nucleotide polymorphisms from the ICY/CY-specific region, confirmed that *C*_*2*_ is located on Chr. 2 and cosegregated with the marker (M7) derived from a non-synonymous single-nucleotide polymorphism of the pentatricopeptide repeat (PPR) gene (*ClPPR*, *Cla97C02G039880*). Additionally, 27 watermelon inbred lines of ICY, CY, and red flesh were evaluated using previously reported *Cllcyb* (*C* locus)-based markers and our *C*_*2*_ locus-linked *ClPPR*-based marker (M7). As a result, dominant alleles at the *C*_*2*_ locus were required to produce CY, in addition to dominant alleles at the *C* locus, while a recessive homozygous genotype at the *C* locus gave the red flesh irrespective of the genotype at the *C*_*2*_ locus. Using a *ClPPR*-based cleaved amplified polymorphic sequence developed in this study and *Cllcyb*-based markers, watermelon cultivars with CY, ICY, and red flesh could be successfully discerned, implying that the combined use of these markers will be efficient for marker-assisted selection of flesh color in watermelon breeding.

## 1 Introduction

Watermelon is a diploid (2n = 2x = 22) vegetative fruit crop belonging to the family Cucurbitaceae and genus *Citrullus*. The global watermelon cultivation area and production are 4,459,239 ha and 161,837,308 tons, respectively, which account for approximately 10% of the major vegetable production (Food and Agriculture Organization, 2020). Watermelon is native to Africa and has been found in temperate and Mediterranean regions outside Africa (Jeffrey, 1975; Paris, 2015). After more than 4,000 years of domestication, wild-type watermelon species with small fruit size, firm flesh, and bitter taste were improved to modern cultivars such as *Citrullus lanatus* (Thunb.) Matsum. & Nakai, which have consumable traits, including the large size, thin rind, and sweet red flesh (Paris, 2015). The whole-genome sequencing (WGS) was completed for ‘97103,’ a Chinese cultivar (Ren et al., 2012; Guo et al., 2013), and ‘Charleston Gray,’ a US heirloom (Wu et al., 2019), which revealed an approximate genome size of 410 Mb and 22,500 genes (Cucurbit Genome Database, CuGenDB, http://cucurbitgenomics.org).

The fruit flesh color of watermelon is categorized as red (scarlet or coral red), yellow (canary, salmon, or pale yellow), orange, and white (UPOV). Different flesh colors result from the accumulation of specific carotenoid components in flesh (Grassi et al., 2013). The accumulation of all-trans-lycopene (lycopene) and xanthophylls (zeaxanthin, neoxanthin, and violaxanthin) in the chromoplasts results in red and canary yellow flesh colors, whereas the accumulation of ξ-carotene and prolycopene or *ß*-carotene results in the salmon yellow and orange flesh colors, respectively (Tadmor et al., 2005; Perkins-Veazie et al., 2006; Bang et al., 2010; Jin et al., 2019). White flesh is resulted from the accumulation of phytofluene, a colorless carotenoid, and light-yellow-colored ξ-carotene (Zhao et al., 2013).

Watermelon flesh color is genetically ascertained by several loci (genes) involved in the carotenoid biosynthesis pathways. According to Henderson (1989), three alleles (*Y*, *y* ^*o*^, and *y*) at the *Y* locus are associated with coral red, orange with a high prolycopene content, and salmon yellow flesh color. Coral red, regulated by locus *Y*, is dominant over the other two colors. Lou (2009) reported that scarlet red and coral red are regulated by the *Y* ^*SCR*^ and *Y* alleles, respectively, and *Y* ^*SCR*^ was dominant over all other alleles of the *Y* locus (*Y* ^*SCR*^ > *Y* > *y* ^*o*^ > *y*). Contrarily, a major QTL on Chr. 1 is responsible for orange flesh with a high *ß*-carotene content, which is dominant to scarlet red flesh (Branham et al., 2017).

The canary yellow flesh (CY) color in watermelon is conferred by the *C* locus, which is dominant over the red (scarlet or coral red) flesh color (Henderson et al., 1996). The lycopene *ß*-cyclase (LCYB) gene of the *C* locus catalyzes the conversion of lycopene to *ß*-carotene, but its mutant allele suppresses LCYB gene function, leading to the accumulation of lycopene and the appearance of red flesh (Bang et al., 2007; Bang et al., 2014). However, Henderson et al. (1998) described that the homozygous recessive *i*-*C* locus results in red flesh irrespective of the presence of the *C* allele, but the gene responsible for *i*-*C* is unknown. In addition, the homozygous recessive *py* locus prevents the formation of canary yellow flesh but develops pale yellow flesh (Bang et al., 2010). In certain genetic backgrounds, red and yellow colors appear as a mixed pattern in the same flesh. Although Bang et al. (2007) reported a complete dominant control for canary yellow flesh, incomplete yellow (mixed with red) flesh appears frequently in F_1_ and segregates in the progeny of crosses between red and canary yellow flesh cultivars, which implies that unknown genetic factors other than the *C* locus may be involved in the determination of canary yellow. Based on our knowledge, there are no research reports on the genetic control of the mixed color pattern in incomplete yellow flesh.

For the marker-assisted selection of fruit flesh color, *LCYB* gene-based markers have been developed to distinguish red from canary yellow (Bang et al., 2007). Jin et al. (2019) also suggested the development of a *CRTISO* gene-based marker for orange flesh with high prolycopene content. Recently, Li et al. (2020) and Lee et al. (2021) described the markers distinguishing scarlet and coral red. However, no markers have been reported for selecting incomplete yellow, which is essential for breeding canary yellow- or red-fleshed watermelons.

Whole-genome resequencing (WGRS) based on next-generation sequencing (NGS) provides comprehensive genome sequence variations that are ideal for identifying DNA markers and genes associated with phenotypic variations (Meuwissen and Goddard, 2010; Saidi and Hajibarat, 2020). The WGRS of a large number of watermelon accessions and genome-wide association study (GWAS) revealed sequence polymorphisms and genes for fruit-related traits, such as fruit shape, flesh color, rind patterns (Park et al., 2018; Subburaj et al., 2019), and disease resistances, including gummy stem blight (Lee et al., 2018). The WGRS of near-isogenic lines successfully revealed a locus related to the high lycopene content in a scarlet red-fleshed watermelon cultivar (Lee et al., 2021).

The present study aimed to identify the genetic control responsible for incomplete yellow flesh in watermelon. Using the WGRS and linkage mapping approach, we identified a novel locus named *C*_*2*_ that controls incomplete yellow flesh, which is required for the development of canary yellow flesh in addition to the previously reported locus *C*. The molecular markers for *C*_*2*_ established in this study will be beneficial for MAS of canary yellow or red-fleshed watermelons.

## 2 Materials and methods

### 2.1 Plant materials and phenotyping of flesh color

**FIGURE 1:**
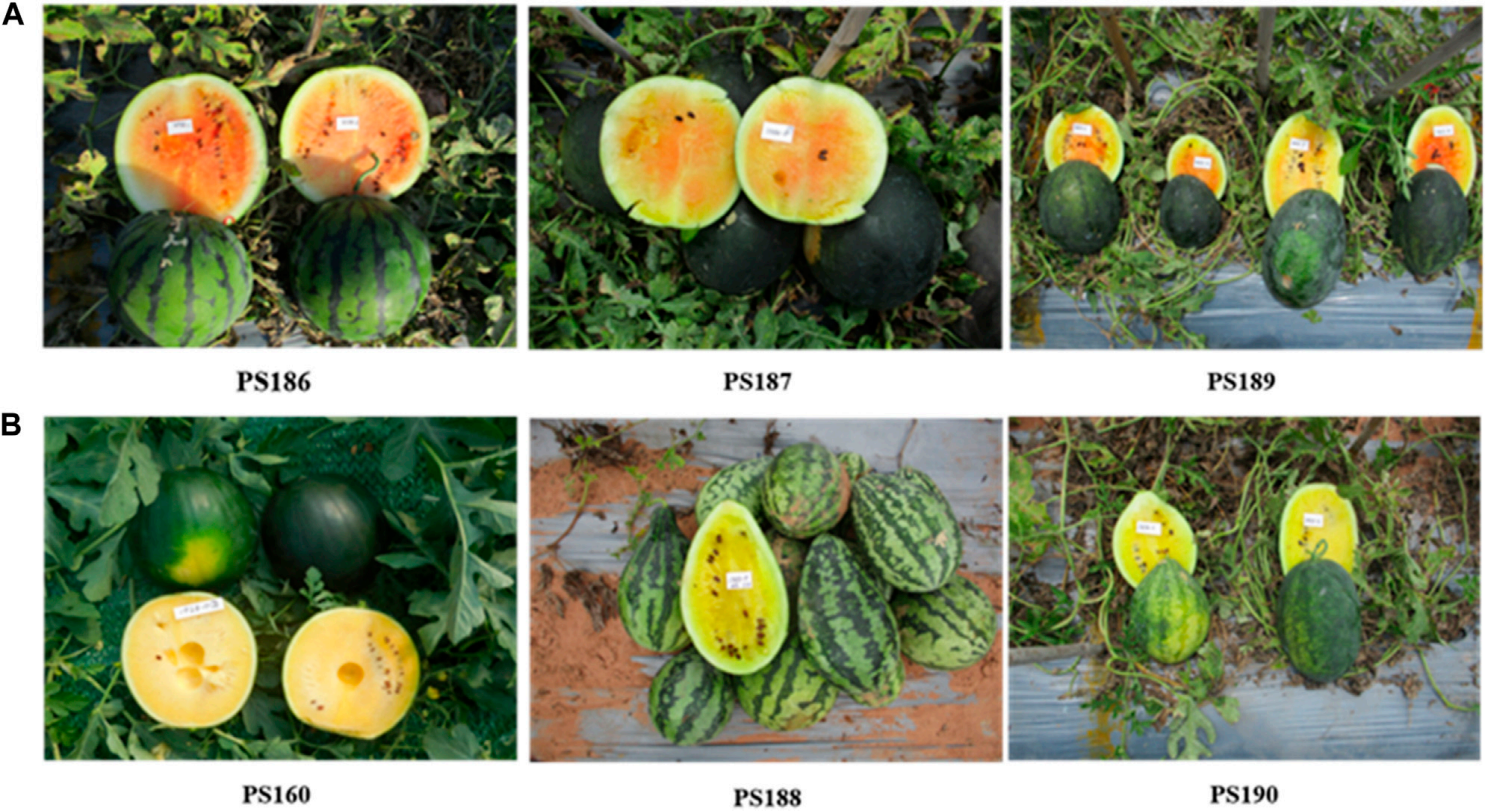
Watermelon inbred lines showing **(A)** incomplete canary yellow flesh color and **(B)** complete canary yellow flash color, which were used for whole-genome resequencing in this study. The ICY group is further divided into severely reddish ICY (ICY-H, PS186 and PS189) and slightly reddish ICY (ICY-L, PS187)-fleshed inbred lines. PS189 and PS190 were used to develop an F_2_ population for genetic mapping of the loci controlling ICY.

In the present investigation, three canary yellow-fleshed inbred lines (CY group: PS160, PS188, and PS190) and three incomplete canary yellow-fleshed (red colored mixed in the yellow background) inbred lines (ICY group: PS186, PS187, and PS189) were used for WGRS (Figure 1). The ICY group is further divided into two severely reddish ICY (ICY-H, PS186 and PS189) and one slightly reddish ICY (ICY-L, PS187) fleshed inbred lines depending on the strength of red color mixed in the yellow background. For genetic inheritance and linkage mapping analysis, an ICY-H inbred line PS189 (maternal parent) was crossed with a CY inbred line PS190 (paternal parent) to produce F_1_ progeny, and then, it was self-pollinated to create a segregating F_2_ population.

Plant cultivation and phenotyping of fruit color were carried out for PS189, PS190, F_1_ hybrids, and F_2_ population in a breeding field of the Partner Seed Company (Gimje, Korea). All seeds were sown in spring, 2021, and seedlings were grafted onto the bottle gourd cultivar ‘Bullojangsaeng.’ Grafted seedlings were planted at 30 cm intervals in a plastic film greenhouse and grown following conventional soil cultivation of a single fruit per plant. For phenotyping, fully matured fruits were harvested on the 40th day after self-pollination, and the center of the fruit was cut in the longitudinal direction. Flesh color was scored and photographed as ICY or CY based on the presence or absence of redness in the flesh.

### 2.2 Whole-genome resequencing

Total genomic DNA was extracted from the first leaf of the seedling for WGRS of the six watermelon inbred lines using the GeneAll Exgene Plant SV mini kit (GeneAll, Seoul, Korea). NGS was performed at Macrogen (Seoul, Korea) by paired-end sequencing (2×, 151 bp) using the HiseqX platform (Illumina, Inc., San Diego, CA, United States).

The raw reads were trimmed to find high-quality clean reads by removing adapter sequences and short reads with a Phred score of <20 and length of <25 bp using cutadapt (v.4.1) (Martin, 2011) and DynamicTrim and LengthSort programs of SolexaQA (v.1.13) (Cox et al., 2010). The cleaned reads were aligned with the watermelon reference genome 97103 v.2 (CuGenDB: http://cucurbitgenomics.org) (Guo et al., 2013) using the BWA (v.0.7.16-r1188) program (Li, 2013). The BAM file created through read mapping was searched for raw sequence variation using the SAMtools (v.0.1.16) program (Li et al., 2009), and the consensus sequence was extracted. An integrated single-nucleotide polymorphism (SNP) matrix between each inbred line was prepared to search for SNPs between the ICY and CY groups (monomorphic between the three inbred lines within each group and polymorphic between the two groups).

A list of unions was constructed using the raw SNP positions obtained by comparing six inbred lines with the reference genome (97103 v.2) as candidates. The SNP loci included in the list were compared with each of the six inbred lines. In the case of missing genotypes at SNP loci within the inbred line, the matrix was completed by imputation from the consensus sequence of the inbred line. The obtained SNPs were classified based on the read rate as homozygous (read rate ≥90%), heterozygous (40%≤ read rate ≤60%), and other (if it could not be distinguished as homozygous or heterozygous). An integrated SNP matrix for each of the six inbred lines was prepared to search for SNPs between the groups of ICY-H, ICY-L, and CY. To identify genomic regions specific to the ICY phenotype, the chromosomal distribution of the SNPs that were monomorphic among the three inbred lines within each group but polymorphic between the two groups was analyzed. Gene annotation information was acquired using watermelon reference genome 97103 v.2.

### 2.3 CAPS marker conversion and genotyping

For the marker conversion of SNPs to a cleaved amplified polymorphic sequence (CAPS), a flanking sequence with a total length of 600 bp was obtained with 300 bp on each side based on the position of each SNP. Restriction enzymes that can differentiate polymorphisms were confirmed using the NEB cutter (v.2.0) program (http://nc2.neb.com/NEBcutter2/index.php). PCR primers for the CAPS were designed based on the franking sequences using the Primer3 (v.0.4.0) program (https://bioinfo.ut.ee/primer3-0.4.0/).

PCR was carried out with 20 µL volume comprising 10 ng of genomic DNA, 1X PCR buffer, 0.1 µM each forward and reverse primer, 0.5 U Taq polymerase, and 0.2 mM dNTPs (Solgent, Daejeon, Korea). The following conditions were used to perform touch-down PCR: 1 cycle at 95°C for 5 min; 10 cycles at 95°C for 15 s, 60°C (decreased by 0.5°C for each cycle) for 30 s, and 72°C for 1 min; and 35 cycles of amplification at 95°C for 15 s, 55°C for 30 s, and 72°C for 1 min. The PCR amplicon was digested using restriction enzymes, in accordance with the manufacturer’s instructions (New England BioLabs Inc., Ipswich, MA, United States). Electrophoresis on a 2% agarose gel at 180 V for around an hour was used to identify the PCR and enzyme-digested products.

### 2.4 Inheritance analysis and construction of the genetic linkage map

The phenotypes of the F_2_ progeny were classified into CY and ICY according to the presence or absence of redness in the fruit flesh. A chi-squared analysis of the flesh color trait segregation ratio in the F_2_ population was performed for Mendelian inheritance using the JoinMap (v.5) program (Kyazma, Wageningen, Netherlands). Furthermore, F_2_ progeny were genotyped by using nine CAPS markers for linkage mapping, and the marker genotypes of each F_2_ individual, which follows PS189 (ICY) and PS190 (CY), and F_1_ were scored as ‘a’, ‘b’, and ‘h’, respectively. The phenotype of each F_2_ individual was scored as ‘a’ for ICY and ‘b’ for CY. The linkage group was created using the JoinMap (v.5) program (Kyazma, Wageningen, Netherlands) based on the Kosambi function and an LOD score of 2.0.

### 2.5 Evaluation of watermelon germplasm

Seeds of a total of 21 watermelon inbreds including 10 CY and 11 red-fleshed lines R were achieved from the Plant Genetics and Breeding Research Center at Pusan National University (Miryang, South Korea) and Partner Seed Company. Genomic DNA was extracted from the first leaf of the seedling using the GeneAll Exgene Plant SV mini kit (GeneAll, Seoul, Korea). Genotyping was performed by using two *LCYB* gene (*C* locus)-based markers (Lcyb and Clcyb.600) (Bang et al., 2007) and a *C*_*2*_ locus-linked marker M7 developed in this study.

## 3 Result

### 3.1 Whole-genome resequencing

Whole-genome sequences were analyzed for three watermelon inbred lines with canary yellow flesh and three with incomplete canary yellow flesh mixed with red, and the sequence data (accession number: PRJNA1002084) were deposited to the GenBank of National Center for Biotechnology Information (https://www.ncbi.nlm.nih.gov/). The number of raw reads produced from each inbred line ranged from 60,736,065 to 77,52,303, with an average of 68,977,937 per line. The total length of the raw reads ranged from 9,171,145,815 bp to 11,705,565,753 bp, with an average of 10,415,668,487 bp. The average Q30, or the ratio of reads with a Phred score of 30 or higher indicating read quality, was 89.44%. The genome coverage, which represents the ratio of the total read length and reference genome size of a watermelon, was in the fold range of 45.58×–55.09×, with an average of 49.01×.

After trimming the raw reads, the average length of the reads ranged from 103.44 to 109.54 bp, with an average of 106.65 bp, and the total length of trimmed reads ranged from 5,689,784,468 bp to 7,133,026,915 bp with an average of 6,389,653,991 bp. The ratio between trimmed reads and raw reads (trimmed/raw (%)) ranged from 58.97% to 63.76%, with an average of 61.43%. After trimming, the range of genome coverage of the six inbred lines was 26.83 × –33.07×, with an average of 30.06 × (Table 1).

**TABLE 1:** Summary of the whole-genome resequencing data for three incomplete canary yellow and three canary yellowfleshed watermelon inbred lines.

Accession	Flesh color^a^	No. of reads	Total length (bp) of reads	Trimmed/raw reads (%)	Genome coverage^b^
PS186	ICY-H	63,234,520	6,757,840,833	61.41	31.49
		63,234,520	6,626,953,198	60.22	
PS187	ICY-L	55,893,932	6,044,434,314	62.41	28.30
		55,893,932	5,983,216,969	61.78	
PS189	ICY-H	61,753,336	6,557,652,550	60.54	30.46
		61,753,336	6,387,887,298	58.97	
PS188	CY	66,636,611	7,133,026,915	60.94	33.07
		66,636,611	6,920,568,150	59.12	
PS160	CY	53,396,134	5,712,481,622	62.29	26.83
		53,396,134	5,689,784,468	62.04	
PS190	CY	58,764,609	6,436,926,252	63.76	30.26
		58,764,609	6,425,075,318	63.64	

^a^ ICY, incomplete canary yellow [ICY-H: severely reddish flesh; ICY-L: slightly reddish fleshed on the strength of red color mixed in the yellow background]; CY, canary yellow.

^b^ Approximate genome coverage (fold) calculated based on the total length of trimmed read.

### 3.2 Identification of the genomic region associated with the flesh color

**FIGURE 2:**
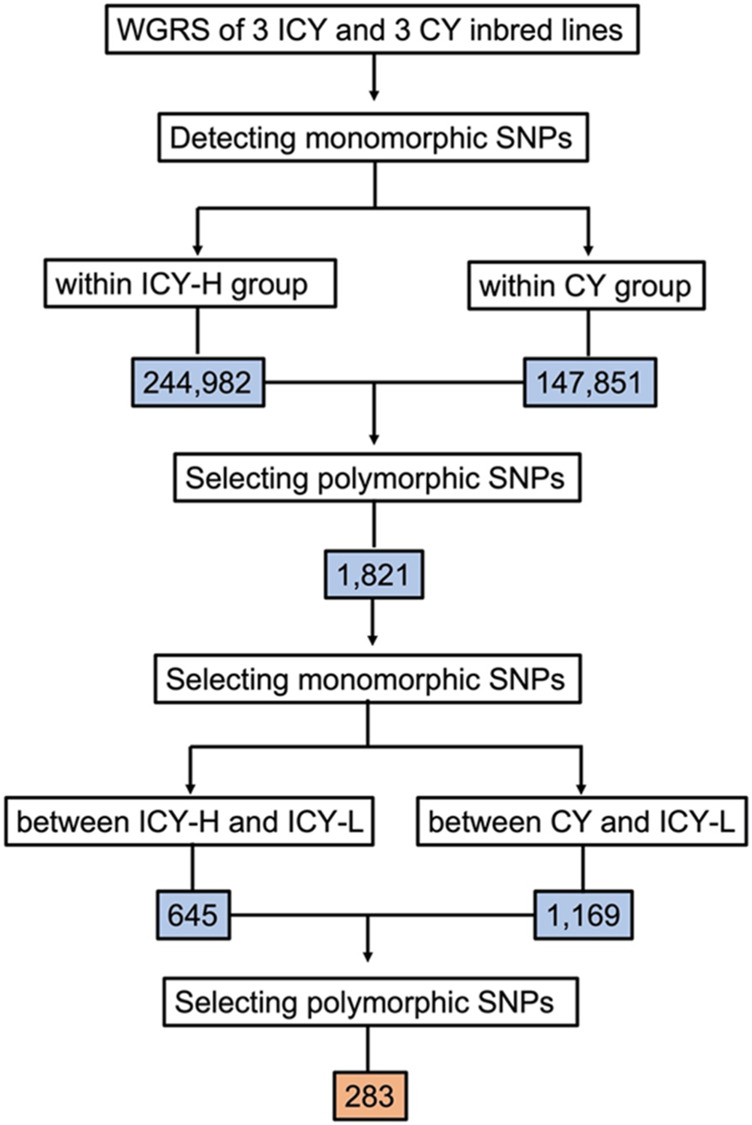
Flow chart showing sequential steps for selecting the single-nucleotide polymorphisms (SNPs) that were polymorphic between incomplete canary yellow-fleshed (ICY group) and canary yellow-fleshed watermelon inbred lines (CY group). WGRS, whole-genome resequencing; ICY-H, severely reddish ICY; and ICY-L, slightly reddish ICY. The number of SNPs is presented in the colored box.

The read mapping of the six inbred lines resulted in an average of 98.48% of all reads being mapped. The number of homozygous SNPs to the reference genome for each inbred line ranged from 154,049 to 189,966, with an average of 174,409 (Table 2). To identify the genomic region for flesh color associated with ICY, we first detected 244,982 and 148,999 SNPs that were monomorphic within the inbred lines of each ICY-H and CY group, respectively. Then, there were a total of 98,602 SNPs that could be compared between the ICY-H and CY groups. Among them, 1,821 homozygous SNPs that were polymorphic between the two groups were selected. Furthermore, among those SNPs, 645 and 1,169 SNPs that were monomorphic between ICY-H and ICY-L groups and between CY and ICY-L groups were selected, respectively. Finally, a total of 283 SNPs that were polymorphic between the two SNP groups (645 vs 1,169 SNPs) were selected as the SNPs specific to ICY and CY (polymorphic regions between ICY and CY groups) (Table 2; Figure 2; Supplementary Table S1).

**TABLE 2:** Statistics of SNP detection of three incomplete canary yellow and three canary yellowfleshed watermelon inbred lines confirmed through mapping to the reference genome.

Accession	Flesh color^a^	No. of total SNPs	No. of homozygous SNPs	No. of heterozygous SNPs	No. of etc^b^
PS186	ICY-H	215,712	181,678	5,185	28,849
PS187	ICY-L	194,128	160,226	5,194	28,708
PS189	ICY-H	226,043	189,966	5,697	30,380
PS188	CY	184,488	154,049	4,643	25,796
PS160	CY	207,203	173,428	5,334	28,441
PS190	CY	233,059	187,107	5,928	30,024
Monomorphic in the ICY-H group			244,982		
Monomorphic in the CY group			147,851		
Polymorphic between the ICY-H and CY groups			1,821		
Monomorphic between ICY-H and ICY-L			645		
Monomorphic between CY and ICY-L			1,169		
Polymorphic between the 645 and 1,169 SNP groups			283		

^a^ ICY, incomplete canary yellow [ICY-H: severely reddish flesh; ICY-L: slightly reddish fleshed on the strength of red color mixed in the yellow background]; CY, canary yellow.

^b^ If it could not have distinguished homozygous and heterozygous SNPs, then it is denoted as etc.

**FIGURE 3:**
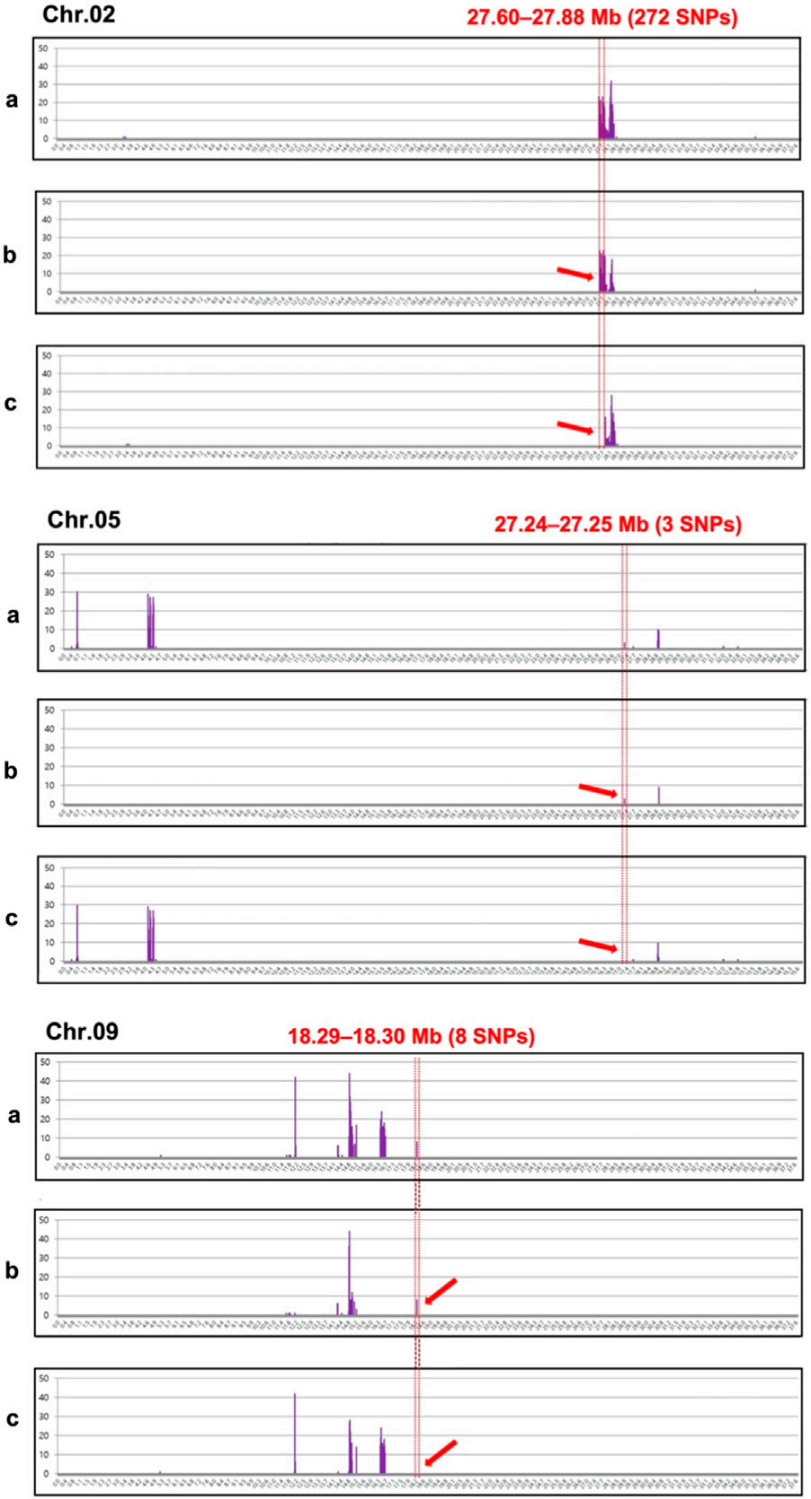
Chromosomal distribution of 283 single-nucleotide polymorphisms (SNPs) showing polymorphisms between the incomplete canary yellow-fleshed watermelon inbred line group (ICY group) and canary yellow-fleshed inbred line group (CY group). The number of SNPs (*Y*-axis) was counted at window sliding of every 4 Mb (*X*-axis) for each chromosome and represented as a vertical bar graph in violet. Three clusters of SNPs (ICY/CY-specific regions) indicating an association with the ICY and CY flesh were detected on Chr. 2, 5, and 9 and depicted using red dotted lines. The red arrows indicate the ICY/CY-specific regions that are delimitated by the red dotted line. **(a)** SNPs that were polymorphic between the severely reddish ICY (ICY-H) and CY group; **(b)** SNPs that were monomorphic between the slightly reddish ICY (ICY-L) and ICY-H group; and **(c)** SNPs that were monomorphic between the ICY-L and CY groups.

Analysis of the chromosomal distribution of 283 SNPs revealed the clusters of SNPs largely on three genomic regions: Chr. 2 (27.60—27.88 Mb, 272 SNPs), Chr. 5 (27.24—27.25 Mb, 3 SNPs), and Chr. 9 (18.29—18.30 Mb, 8 SNPs) (Figure 3). The majority of these SNPs, specifically 272 out of 283, are densely clustered within the 27.60–27.88 Mb genomic region on Chr. 2. Additionally, two other notable but smaller clusters can be observed on Chr. 5 between the 27.24–27.25 Mb genomic region with three SNPs and on Chr. 9 from 18.29–18.30 Mb with eight SNPs (Figure 3). In the region of Chr. 2, 272 SNPs were recorded in both genic and intergenic regions. A total of 14 genes were annotated in the genomic region of Chr. 2 with 37 SNPs. Among these SNPs, 29 were located in the intron, while eight were in the exon (Supplementary Table S1). Of the eight SNPs identified in the exon region, five non-synonymous SNPs were found in five genes (Table 3; Supplementary Table S2). A total of 17 indels were found in 11 genes, all of which were located in the intron region (Supplementary Table S2). However, there were no genes with variations in the corresponding region of Chr. 5 and 9, but three and eight SNPs, respectively, were found in the intergenic region.

**TABLE 3:** List of annotated genes with significant SNPs in the polymorphic region between the incomplete canary yellow-fleshed watermelon inbred lines group (ICY group) and canary yellow-fleshed inbred line group (CY group) of Chr. 2

Gene ID	Gene description	SNP^a^	SNP position
*Cla97C02G039770*	Protein FAR1-RELATED SEQUENCE 5	A/C*	27,683,685
*Cla97C02G039790*	Exosome complex component RRP41 homolog	G/A*	27,691,110
*Cla97C02G039830*	E3 ubiquitin ligase BIG BROTHER-related	C/T*	27,722,913
*Cla97C02G039880*	Pentatricopeptide repeat	G/C*	27,789,237
*Cla97C02G039900*	Myosin-1-like	A/G	27,819,065
*Cla97C02G039920*	F-box/LRR-repeat 4-like protein	T/C	27,836,687
*Cla97C02G039950*	GRAM domain-containing protein	T/C*	27,858,223
*Cla97C02G039960*	Transcription factor DIVARICATA	C/T	27,864,646

^a^ Mutation type of non-synonymous SNPs is denoted by *, and the rest were synonymous.

### 3.3 Genetic inheritance of incomplete canary yellow flesh color

**FIGURE 4:**
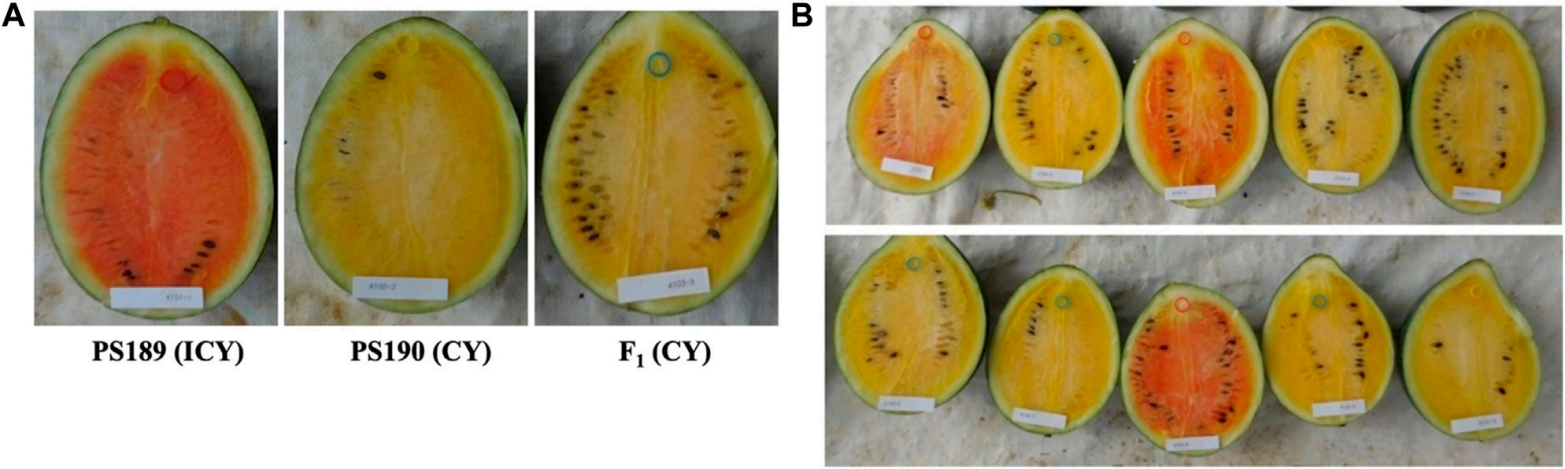
Fruit flesh color of **(A)** inbred lines PS189 and PS190 and their F_1_ and **(B)** F_2_ plants. Segregating for flesh color: ICY, incomplete canary yellow; CY, canary yellow.

The F_1_ progeny from the cross between PS189 (ICY-H) and PS190 (CY) showed a CY flesh color that was close to that of PS190 (Figure 4). In the F_2_ population of 135 individuals, 94 showed CY flesh color and 41 exhibited ICY flesh color, which was consistent with the Mendelian ratio of 3:1 (X^2^ = 2.076, *p*-value = 0.149). The fruit flesh color of the F_1_ plants and their segregation ratio in the F_2_ population indicated that CY was dominant over ICY and was regulated by a single locus. The locus was named *C*_*2*_ in this study.

### 3.4 Genetic linkage mapping of the *C*_*2*_ locus

**FIGURE 5:**
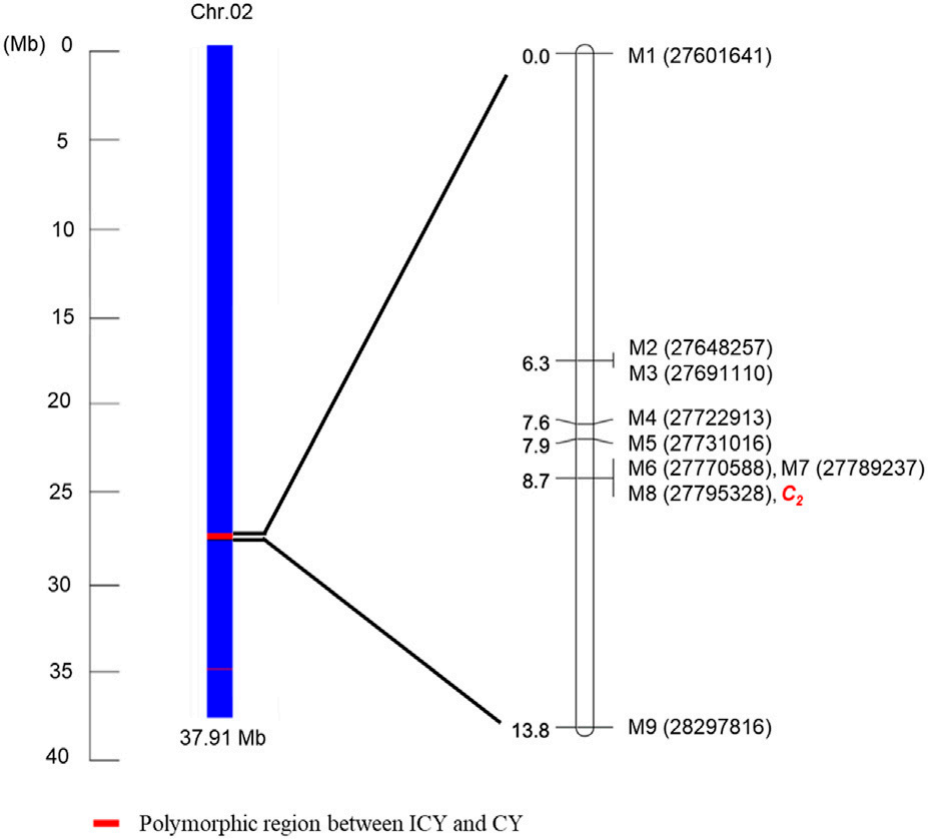
Schematic representation of Chr. 2 showing the ICY/CY-specific region [polymorphic region (red) between the incomplete canary yellow-fleshed watermelon inbred line group (ICY group) and canary yellow-fleshed inbred line group (CY group)]. A genetic linkage map was constructed using seven cleaved amplified polymorphic sequence markers developed from the SNPs (Table 5) in the ICY/CY-specific region, two CAPS markers developed from the SNPs flanking the ICY/CY-specific region, and *C*_*2*_ locus controlling ICY and CY in an F_2_ population.

**FIGURE 6:**
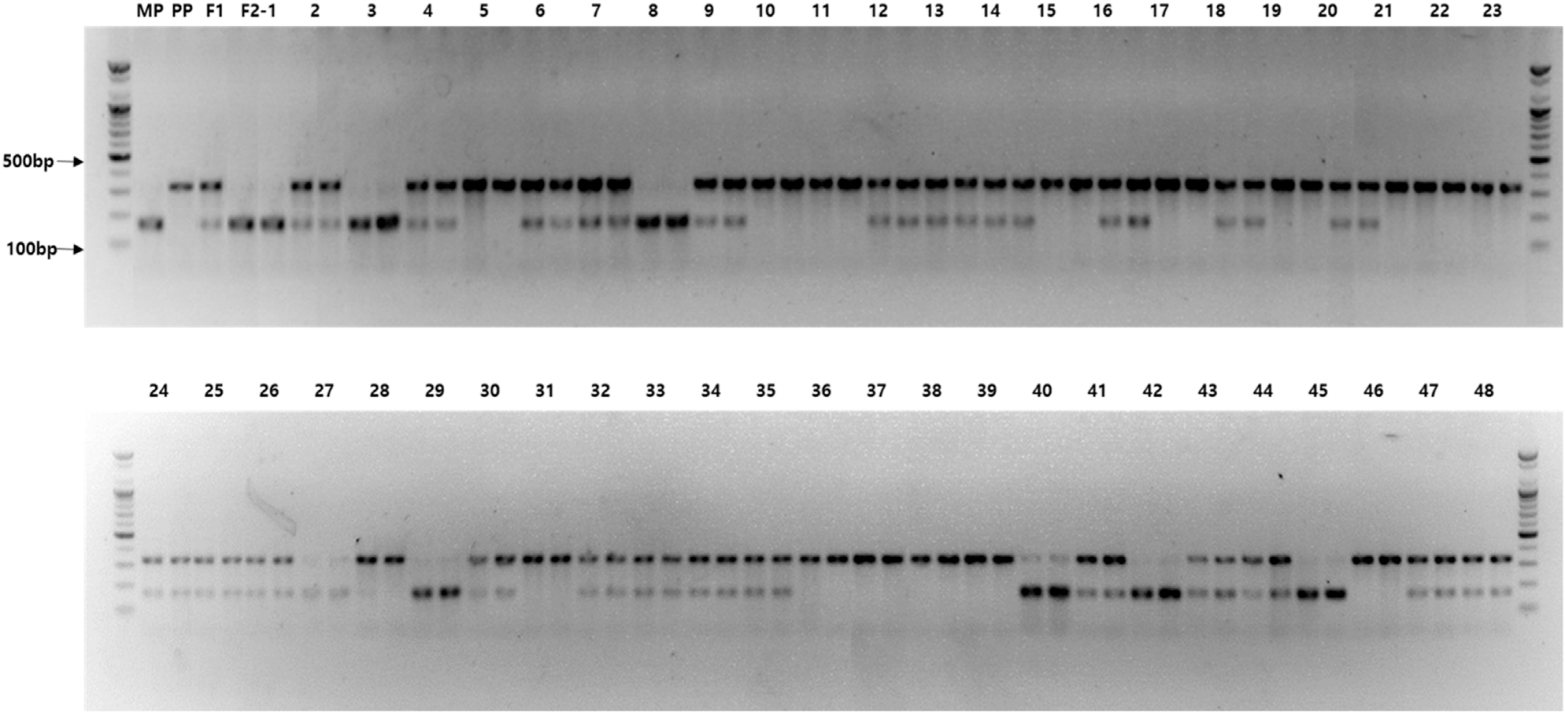
Agarose gel image exhibiting the genotyping results for the *Cla97C02G039880 (ClPPR)* gene-based cleaved amplified polymorphic sequence (M7) that were cosegregating with the incomplete canary yellow (ICY) and canary yellow (CY) flesh color in F_2_ watermelon plants. M, 100 bp size marker; MP, maternal parent (PS189); PP, paternal parent (PS190); F_1_, progeny from cross between MP and PP; and F_2_—1–48, F_2_ plants (two PCR replicates).

For genetic linkage map construction, the cleaved amplified polymorphic sequence (CAPS) markers (M1 to M9) were designed based on nine SNPs located within the region of Chr. 2 polymorphic between ICY and CY groups (ICY/CY-specific region) (Table 4). Of the nine SNPs used for marker development, four were located in the genic region and two were a missense mutation (non-synonymous), causing amino acid sequence substitution. The remaining five SNPs were in intergenic regions. These CAPS markers were genotyped on 135 F_2_ plants (Supplementary Table S3), and a single linkage group (13.5 cM) composed of nine CAPS markers and the *C*_*2*_ locus was constructed (Figure 5). In this linkage group, all CAPS markers were mapped in accordance with their physical genomic orders and the *C*_*2*_ locus was cosegregating with three markers M6, M7, and M8. Among these three cosegregating markers, M7 (Figure 6) was a non-synonymous SNP-based marker for the gene (*Cla97C02G039880*) encoding pentatricopeptide repeat protein (PPR). These results confirmed that the *C*_*2*_ locus exists in the ICY/CY-specific region of Chr. 2, and *Cla97C02G039880 (ClPPR)* is a putative candidate gene for *C*_*2.*_

**TABLE 4:** Information of cleaved amplified polymorphic sequence markers designed from the single-nucleotide polymorphisms in the region of Chr. 2 (27.60–28.29 Mb) polymorphic between incomplete canary yellow and canary yellow flesh in watermelon.

Marker^a^	Type^b^	SNP^c^	SNP position	Enzyme	Gene ID	Gene description	Primer sequence (5′-3′)		Genetic distance (cM)	No. of recombinants
							Forward	Reverse		
M1*	CAPS	C/T	27,601,641	*Xmn*I	Intergenic	-	GGG​GAT​ATA​AAG​CTC​AAT​GAG​AAA	CTC​TAC​TTC​CAA​CAT​CCT​CCA​AAT	0.0	11
M2	CAPS	G/A	27,648,257	*HpyCH4*V	Intergenic	-	TTT​CAC​TCA​CAT​GTC​TTT​ACC​TGA	TGT​TAG​GGA​GTG​TTC​GGA​GAA	6.3	2
M3	CAPS	G/A*	27,691,110	*Nsi*I	*Cla97C02G039790*	Exosome complex component RRP41 homolog	AGG​CCT​CAT​TTG​CCT​TCT​TT	TTG​CGA​ACA​ATG​CAA​CAT​TT	6.3	3
M4	dCAPS	C/T*	27,722,913	*Sca*I	*Cla97C02G039830*	E3 ubiquitin ligase BIG BROTHER-related	AGG​TCT​GCC​CTG​TCT​GCA​GTA​C	CCC​ACC​ATA​CAC​ATT​TTC​CA	7.6	1
M5	CAPS	A/G	27,731,016	*Dra*I	Intergenic	-	TCC​TTT​CTT​TTG​ATC​TTG​AGT​TGA	ATC​CAA​CTC​ATG​TAC​ACC​CCT​AAT	7.9	1
M6	CAPS	G/A	27,770,588	*Fok*I	Intergenic	-	TTT​CCT​CTT​TCT​TTC​ATT​CAA​ACC	TTT​TAA​ACC​CTT​CCA​AAC​CTA​TTT​T	8.7	0
M7	CAPS	G/C*	27,789237	*Mnl*I	*Cla97C02G039880*	Pentatricopeptide repeat	GCT​CGG​GTG​TTC​ATT​GTC​TT	CCG​CAG​TGG​ATG​TTA​ATC​CT	8.7	0
M8	CAPS	G/A	27,795,328	*Taq*I	Intergenic	-	TGA​TAG​GAC​TGA​CTC​CAC​TTT​TCA	AAA​TTG​GAG​GGA​ACC​ACC​TT	8.7	0
M9*	CAPS	G/T	28,297,816	*Fok*I	*Cla97C02G040330* (intron)	Transmembrane protein	TAA​TCA​ATC​AAA​TCC​CAA​TGT​CC	GCT​ATG​CAA​CAG​ATT​CAG​ACA​CTT	13.8	5

^a^ Two markers that are derived from the SNPs flanking the region of Chr. 2 (27.60–28.29 Mb) are denoted by *.

^b^ dCAPS, derived cleaved amplified polymorphic sequence.

^c^ Mutation type of non-synonymous SNPs is denoted by *.

### 3.5 Marker evaluation for watermelon accessions

A total of 28 watermelon accessions, including 13 inbred lines with CY, 11 with red fruit flesh, and PS186, PS187, and PS189 with ICY, were genotyped using the M7 marker and two previous LCYB gene (*C* locus)-based markers, Lcyb and Clcyb.600, discerning CY and red flesh color (Bang et al., 2007; Bang et al., 2014) (Table 5). As a result, all CY lines showed the dominant homozygous genotype for all LCYB gene-based markers (*CC*) and the *C*_*2*_-linked M7 (*C*_*2*_ *C*_*2*_). In contrast, the accessions with red flesh showed either dominant homozygosity or recessive homozygosity marker genotype (*c*_*2*_ *c*_*2*_) for *C*_*2*_-linked markers, but all showed recessive homozygosity (*cc*) for the two *LCYB* gene-based markers. For PS186, PS187, and PS189 with ICY, *LCYB* gene-based markers were genotyped as dominant homozygous, whereas the *C*_*2*_-linked markers were genotyped as recessive homozygous. Based on the results, it is likely that dominant alleles at both the *C* locus and the *C*_*2*_ locus are required to produce canary yellow flesh, while a recessive homozygous genotype at the *C* locus will give the red flesh irrespective of the genotype at the *C*_*2*_ locus. This suggests that the recessive allele at the *C*_*2*_ locus (*c*_*2*_) is in an epistatic relationship over the *C* locus gene, meaning that the effect of the *LCYB* gene for canary yellow flesh is partially masked by *c*_*2*_.

**TABLE 5:** Evaluation of 27 watermelon inbred lines using *Cllcyb* gene (*C* locus)-based markers and *C*_*2*_ locus-linked *ClPPR* gene-based marker.

Accession	Phenotype^a^	Genotype of the *Cllcyb*-based marker^b^		Genotype of the *ClPPR-*based marker
		Lcyb	Clcyb.600	M7
PS186	ICY	*CC*	*CC*	*c*_*2*_ *c*_*2*_
PS187	ICY	*CC*	*CC*	*c*_*2*_ *c*_*2*_
PS189	ICY	*CC*	*CC*	*c*_*2*_ *c*_*2*_
PS160	CY	*CC*	*CC*	*C*_*2*_ *C*_*2*_
PS188	CY	*CC*	*CC*	*C*_*2*_ *C*_*2*_
PS190	CY	*CC*	*CC*	*C*_*2*_ *C*_*2*_
2901	CY	*CC*	*CC*	*C*_*2*_ *C*_*2*_
2902	CY	*CC*	*CC*	*C*_*2*_ *C*_*2*_
2903	CY	*CC*	*CC*	*C*_*2*_ *C*_*2*_
2904	CY	*CC*	*CC*	*C*_*2*_ *C*_*2*_
2906	CY	*CC*	*CC*	*C*_*2*_ *C*_*2*_
2908	CY	*CC*	*CC*	*C*_*2*_ *c*_*2*_
2909	CY	*CC*	*CC*	*C*_*2*_ *C*_*2*_
2911	CY	*CC*	*CC*	*C*_*2*_ *C*_*2*_
ALDF	CY	*CC*	*CC*	*C*_*2*_ *C*_*2*_
OTO9491	CY	*CC*	*CC*	*C*_*2*_ *C*_*2*_
2962	R	*cc*	*cc*	*C*_*2*_ *C*_*2*_
2964	R	*cc*	*cc*	*c*_*2*_ *c*_*2*_
2966	R	*cc*	*cc*	*c*_*2*_ *c*_*2*_
2985	R	*cc*	*cc*	*C*_*2*_ *C*_*2*_
2987	R	*cc*	*cc*	*c*_*2*_ *c*_*2*_
2995	R	*cc*	*cc*	*C*_*2*_ *C*_*2*_
2998	R	*cc*	*cc*	*c*_*2*_ *c*_*2*_
3005	R	*cc*	*cc*	*c*_*2*_ *c*_*2*_
DAH	R	*cc*	*cc*	*c*_*2*_ *c*_*2*_
JB11-3	R	*cc*	*cc*	*c*_*2*_ *c*_*2*_
JB38-1	R	*cc*	*cc*	*C*_*2*_ *C*_*2*_

^a^ ICY, incomplete canary yellow; CY, canary yellow; R, scarlet or coral red.

^b^ *CC*, LCYB-gene-based marker genotype for the homozygous wild-type allele; cc, LCYB-gene-based marker genotype for the homozygous mutant-type allele.

*C*_*2*_ *C*_*2*_, *C*_*2*_ locus-associated marker genotype for the homozygous wild-type allele; c_2_c_2_, *C*_*2*_ locus-associated marker genotype for the homozygous mutant-type allele.

## 4 Discussion

Differences in the flesh color of watermelons are mostly due to the composition and amount of carotenoids. The selection of specific flesh color is an important breeding target in terms of the consumer’s preferences and nutritional aspects. Previous studies have suggested that those genes involved in the metabolism of carotenoids are associated with various colors of watermelon flesh. The canary yellow flesh (CY) is regulated by the dominant *C* locus, and a homozygous recessive mutation of *C* results in red (scarlet and coral red) flesh (Bang et al., 2010). Molecular studies have characterized the *C* locus as the gene *Cllcyb* (*Cla97C04G070940*) encoding *LCYB* (Wang P. et al., 2019; Wang C. et al., 2019; Zhang et al., 2020), and gene-based markers have been used for the MAS of canary yellow and red flesh. However, some watermelon inbred lines with the *Cllcyb* marker genotype of dominant homozygosity (*CC*) have been found to show a mixture of CY and red flesh (incomplete canary yellow, ICY). Although it has been suggested that environmental variables might contribute to ICY (Bang et al., 2007), no genetic factors that are involved in the expression of ICY are currently known.

In this study, to identify possible genetic factors determining ICY and CY, we compared genome sequences of six ICY and CY inbred lines to identify genomic regions specific to this trait (ICY-specific genomic region) and mapped this region using an F_2_ population. A whole-genome sequence comparison of the ICY and CY inbred groups identified a genomic region containing 66 genes on Chr. 2 that was polymorphic between these groups. Genetic inheritance analysis using an F_2_ population indicated that ICY is controlled by the homozygous recessive alleles at a single locus *C*_*2*_, named in this study. Genetic linkage mapping using a set of CAPS markers derived from the SNPs in the ICY-specific genomic region showed that these markers are tightly linked to *C*_*2*_. In this map, a CAPS (M7) derived from a non-synonymous SNP in the gene encoding PPR cosegregated with the flesh color of 135 F_2_ plants, possibly implying that *ClPPR* (*Cla97C02G039880*) may be a putative candidate gene for *C*_*2*_. Furthermore, genotyping of 28 watermelon accessions using the two *Cllcyb* gene-based markers and *ClPPR*-based CAPS markers (M7) indicated that all CY inbreds require dominant alleles for both *C* and *C*_*2*_ and that *c*_*2*_ is dominantly epistatic to *C*.

Bang et al. (2010) identified that the pale yellow flesh color in watermelon is due to a locus distinct from *i-C*, indicating the involvement of at least two loci in the variation of canary yellow, pale yellow, and red flesh. This resonates with our findings on the influence of gene interactions in the expression of ICY. Bang et al. (2010) also found that the expression of pale yellow flesh required a dominant allele at the *C* locus, analogous to the necessity for that allele in the expression of ICY, with the presence of a necessary allele at the *C*_*2*_ locus. We observed that all CY plants demonstrated dominant homozygosity or heterozygosity for both the *LCYB* and the *C*_*2*_ locus (*PPR* gene). However, the presence of a recessive homozygosity at the *C*_*2*_ locus (*c*_*2*_ *c*_*2*_) indicates an epistatic relationship over the *C* locus gene, partially masking the effect of the *LCYB* gene for canary yellow flesh. In essence, our study is consistent with the multi-gene influences and epistatic relationships found by Bang et al. (2010), highlighting the complex gene interactions influencing watermelon flesh color.

PPRs are a diverse group of proteins predominantly present in plants (Lurin et al., 2004), play crucial roles in organelle formation, plant growth and development (Sun et al., 2018), and expression of cytoplasmic male sterility (CMS) (Jo et al., 2016), and affect fruit development and flesh color formation (Eriksson et al., 2004). Moreover, PPRs are linked to the early chloroplast formation and plastid gene expression (Pyo et al., 2013). Protein deficiency of PPR generally caused embryo development delay in maize (Sun et al., 2019; Yuan et al., 2019), abnormal chloroplast development, and aberrant pigmentation in seeds in *Arabidopsis* (Bryant et al., 2011). The significance of PPR proteins lies in their role as essential proteins that bind to RNA and can regulate gene expression in multiple post-transcriptional processes, including shearing, RNA editing, splicing, degradation, and translation in the chloroplasts (Wang et al., 2021a), plastids (Wang et al., 2021b), and mitochondria (Binder et al., 2013). The PPR protein family has been categorized into two subfamilies, namely, P type and PPR-like long and short (PLS) type (Lurin et al., 2004). Several studies have demonstrated that PPR proteins belonging to these subfamilies, such as the *P*-type PPR protein ACM1 (Wang et al., 2021b) and the *PLS*-type PPR protein MORF9 (Yan et al., 2017), have an impact on plastid development. Some *PPR* members in the *P*-subfamily of watermelon (*ClPPR*) had high expression levels in the fruit flesh, fruit rind, or both rind and flesh tissues, indicating that they might be crucial in coordinating the development of flesh and rind in watermelons (Subburaj et al., 2020). In contrast, the majority of subgroups in the PLS-subfamily showed preferential expression in the rind; high expression levels were observed at all fruit development stages for rind, but only at 10-day post anthesis (DPA) for flesh. Subburaj et al. (2020) suggested that these PLS-subfamily genes may play significant roles in the fruit rind and early fruit flesh development of watermelon. Taken together, these outcomes suggest that the *ClPPR* genes are likely involved in fruit development of watermelon including chloroplast-to-chromoplast transition in fruit flesh (Subburaj et al., 2020).

Subburaj et al. (2020) identified and characterized 422 *ClPPR* genes in the watermelon genome, and out of them, four *ClPPR* genes are associated with orange, two genes with yellow, and one gene with red flesh color among marker genotypes and phenotypes of orange, yellow, and red flesh color in all surveyed lines. Various studies suggested that *ClaPPR11* co-localized with *ß*-carortene-related QTL on Chr.1 (Branham et al., 2017), whereas *ClaPPR140* co-localized with lycopene-related QTL on Chr.4 (Liu et al., 2015). However, *ClaPPR25* and *ClaPPR95* were not co-localized with any QTL for flesh color. Similarly, another PPR member *ClPPR* (*Cla97C06G122120*) on Chr. 6 has been also reported as a candidate gene for the locus *Cyf* that regulates the carotenoid accumulation level in watermelon (Liu et al., 2023). The mutant allele of *Cyf* was responsible for pale yellow flesh (Liu et al., 2023). However, the molecular mechanism for genetic control of the PPR on fruit flesh color formation is not yet fully elucidated. PPR proteins have so far been discovered to be cis-element-recognizing site-specific RNA factors in the plastid (Okuda et al., 2006). PPR (CRR4) specifically those nucleotide sequences nearby cis-acting element of the ndhD-1 site, allowing the scaffold for RNA editing (conversion of C to U) and likely making it easier for the putative RNA editing enzyme to access these sequences (Okuda et al., 2006). The deficiency of a single PPR protein can have an impact on RNA editing at multiple locations in the gene, consequently influencing gene expression within the plastid (Okuda et al., 2006). In a recent study by Galpaz et al. (2018), it was found that *CmPPR1* participates in RNA editing processes within plastids, which are responsible for the accumulation of carotenoids and chlorophyll, consequently giving rise to white, orange, and green flesh formation in melon. It has also been suggested that the *CmPPR1* gene might be involved in retrograde signaling from plastids to the nucleus, influencing the expression of plastid-targeted genes, exhibiting that PPR proteins are involved in the variation of fruit flesh color (Galpaz et al., 2018).

Plastids can be categorized into three groups based on their pigments: chloroplasts, chromoplasts, and leucoplasts. The development of chloroplasts through the involvement of PPR proteins has been reported by numerous studies (Bryant et al., 2011; Pyo et al., 2013). The *ClPPR* gene might be involved in chromoplast development, carotenoid accumulation, and flesh color regulation as well (Liu et al., 2023). However, there is limited research available on the role of PPR proteins in chromoplast development and the formation of flesh color in watermelon. Notably, there are noticeable differences between globular and crystalloid chromoplasts in terms of their structure and color. The presence of red flesh in watermelon is primarily associated with the presence of crystalloid plastid structures, whereas white, yellow, or orange flesh indicates the presence of globular chromoplasts (Fang et al., 2020). These structural differences are likely attributed to variations in pigment components. In a study by Fang et al. (2020), a direct relationship was observed between the formation of crystalloid chromoplasts and lycopene accumulation. Conversely, excessive accumulation of violaxanthin, lutein, *γ*-carotene, and *ß*-carotene led to the development of globular chromoplasts, and they have a potential role in the regulation of carotenoid accumulation and flesh color. The chromoplast’s plastoglobulus serves as a specialized structure responsible for the mechanism of producing and storing carotenoids (Brehelin and Kessler, 2008). The *PPR* gene may specifically influence the number of plastoglobuli, thereby contributing to differences in flesh color. Fang et al. (2020) observed a progressive increase in the accumulation of carotenoids during the fruit flesh development of pale yellow-fleshed ‘COS’ and canary yellow-fleshed ‘PI 635597’watermelon varieties. This increase in carotenoid accumulation was accompanied by an increase in both the quantity and size of chromoplasts and plastoglobuli. Importantly, they reported that the size and number of plastoglobuli were significantly greater in PI 635597 than those in COS, suggesting that the enlarged size and number of plastoglobuli may contribute to the formation of canary yellow flesh color.

In conclusion, we identified a novel locus, *C*_*2*_, which is required for CY flesh color in watermelon in addition to the previously known *C* locus cloned as the *Cllcyb*. Our genetic mapping results indicated that *C*_*2*_ is located on Chr. 2, and *ClPPR* (*Cla97C02G039880*) encoding a member of PPR protein is a putative candidate gene for the locus. Using a *ClPPR*-based CAPS developed in this study and *Cllcyb*-based markers, watermelon cultivars with CY, ICY, and red flesh could be successfully discerned, implying that the combined use of these markers will be efficient for MAS of flesh color in watermelon breeding. Nevertheless, the molecular mechanism for the involvement of *ClPPR* in carotenoid synthesis and its interaction with the *Cllcyb* remains to be elucidated.

## Data Availability

The WGRS datasets presented in this study can be found in online repositories. This data can be found here: National Center for Biotechnology Information (NCBI) (https://www.ncbi.nlm.nih.gov/), accession number PRJNA1002084.
